# Whole-Genome Analysis of *Bacillus paranthracis* Qf-1 Isolated from Mink (*Neogale vison*)

**DOI:** 10.3390/microorganisms13092106

**Published:** 2025-09-09

**Authors:** Haotian Cai, Yao Chen, Xiaoyang Wu, Xibao Wang, Yongquan Shang, Qinguo Wei, Weilai Sha, Honghai Zhang

**Affiliations:** College of Life Sciences, Qufu Normal University, Qufu 273100, China; caihaotian0915@163.com (H.C.); chenyao@qfnu.edu.cn (Y.C.); wuxiaoyang1988@126.com (X.W.); wangxibao1995@163.com (X.W.); yongquanshang@163.com (Y.S.); qgwei2008@163.com (Q.W.); shaweilai@163.com (W.S.)

**Keywords:** *Neogale vison*, *Bacillus paranthracis* Qf-1, culturomics, genome, pathogenicity

## Abstract

*Bacillus paranthracis*, a species of the genus *Bacillus*, is a Gram-positive bacterium classified as an opportunistic pathogen that can cause foodborne diarrhea and other intestinal diseases in humans and various animals. To date, there has been limited research on *B. paranthracis*, and there are few records of this bacterium being isolated from animal intestines. In this study, a strain named Qf-1 was isolated and purified from faecal samples of mink. Through culturomics, 16S rRNA gene sequencing, whole-genome sequencing, and average nucleotide identity (ANI) analysis, the strain was confirmed to be *B. paranthracis*. Whole-genome sequencing revealed that the strain has a genome size of 5.27 Mb, comprising one chromosome (5,224,739 bp) and one plasmid (51,964 bp). Functional annotation of its genome identified multiple potential pathogenic factors associated with pneumonia, including the key genes *AsbD* and *AsbF*, which facilitate bacterial colonisation of the lungs and trigger inflammatory responses, as well as *EsxB* and *EsxL*, which exacerbate lung inflammation and promote infection spread. Comparative genomics analysis revealed that this strain shares a close evolutionary relationship with previously reported *B. paranthracis* strains. The structure and function of the bacterial genes were analyzed in depth using multi-omics methods. Through mouse pathogenicity experiments, it was found that this bacterium may cause pneumonia and enteritis in mice. We predict that it may also pose a threat to the health of the mink. These research findings contribute to the establishment of a stable experimental model between pathogens and mink hosts, laying the foundation for further elucidating their pathogenicity and pathogenic mechanisms. This is of great significance for the diagnosis and prevention of bacterial diseases in mink in the future.

## 1. Introduction

The mink (*Neogale vison*), a mammal of the order Carnivora, family Mustelidae, genus Mustela, is a species of ecological importance and economic value [1]. The main mink-breeding countries in the world currently include Denmark, China, the United States, Canada, and Russia. In China, Shandong Province is the largest mink-skin-producing province, accounting for 57.07%, followed by Liaoning Province, which accounts for 33.29%. Although mink farming can bring huge economic benefits, the lack of unified mink-breeding requirements and standards has led to the occurrence and spread of various mink bacterial diseases and viral infections, which seriously endanger the healthy development of the mink-breeding industry worldwide [2,3]. In addition, minks play an important role in the spread of animal-source diseases and emerging diseases. Their intestines contain a variety of important zoonotic pathogens [4]. Currently, research on viral diseases in mink has become increasingly in-depth and systematic, with significant progress achieved in several key areas. For example, scientists have gained a clearer understanding of fundamental issues such as the taxonomic status, genomic structure, gene function, and pathogenic mechanisms of mink viruses, and have used this knowledge to advance applied research such as vaccine development. In contrast, research on bacterial diseases in mink has lagged behind, particularly in terms of understanding pathogenic mechanisms and developing control measures, which require further exploration. With the development of mink farming, the incidence of bacterial epidemics has been increasing, causing irreversible damage to the industry. In recent years, the pathogenic mechanisms of bacterial diseases in mink and ecological control measures have increasingly attracted research attention. According to current research, various pathogenic bacteria can cause respiratory, digestive, and skin infections in mink, such as *Escherichia coli*, *Klebsiella pneumoniae*, *Pseudomonas aeruginosa*, *Streptococcus canis*, *Streptococcus dysgalactiae*, and *Staphylococcus delphini*, among others [5,6,7,8]. These pathogens pose a potential threat to the economic animal husbandry industry, and their pathogenic mechanisms and control strategies require further investigation [9]. In summary, the isolation and identification of pathogenic bacteria are of great significance for the development of new antimicrobial drugs and the optimization of vaccine strategies. This process lays an important foundation for in-depth research into the pathogenic mechanisms and drug resistance mechanisms of bacteria, and is a key step in achieving precise prevention and control of bacterial diseases in mink.

The *Bacillus cereus* group is a cluster of highly phylogenetically related species of spore-forming bacteria [10], which are widely distributed in various environments. In addition, Liu proposed in 2017 that nine new Gram-positive bacteria, including *Bacillus paranthracis*, belong to the *Bacillus cereus* group [11]. This marks the first isolation and identification of the *B. paranthracis* strain. *Bacillus paranthracis*, a species of the genus *Bacillus*, is a Gram-positive bacterium classified as an opportunistic pathogen that can cause foodborne diarrhea and other intestinal diseases in humans and various animals. Up to now, strains of *B. paranthracis* have been isolated from various sources. This includes a blood sample from a patient who died of Ebola virus disease (EVD) [12], pet reptiles [13], water sources [14], the surface of book pages [15], and the human intestine [16]. In addition, according to research, *B. paranthracis* can cause food-borne diarrhea and emesis in humans [17]. In 2024, a food poisoning outbreak caused by *B. paranthracis* occurred in Huzhou, China [18]. The source of the outbreak was rice contaminated with this bacterial strain. *B. paranthracis* is widely present in various environments, and over time, it will be isolated from an increasing number of environments in its pathogenic form, affecting the health of humans and other animals. The isolation and identification of *B. paranthracis* not only lay the foundation for in-depth research into its pathogenic mechanisms but also provide a theoretical basis for the healthy and green farming of economic animals, thereby improving economic efficiency. Furthermore, this may provide a solid scientific basis for early warning of zoonotic diseases. Therefore, the isolation, identification, and pathogenicity studies of *B. paranthracis* remain fundamental and indispensable scientific endeavors.

In summary, the isolation and identification of pathogenic bacteria are crucial for the prevention and control of epizootic diseases in minks. This study aims to conduct a comprehensive analysis of the strain *B. paranthracis* Qf-1 isolated from minks. Through culturomics, 16S rRNA gene sequencing, whole-genome sequencing, and comparative genomic analysis, we investigated the genetic characteristics and functions to explore its potential pathogenicity. The results not only supplemented the biological data of this strain and explored its pathogenicity but also provided a scientific basis for the formulation of prevention and control strategies for bacterial diseases in mink.

## 2. Materials and Methods

### 2.1. Sample Collection

On 30 November 2024, fecal samples from minks with pneumonia were collected at the Youan Mink Breeding Co., Ltd., located in Qingdao, Shandong Province, China (36°10′34.02″ N, 119°59′15.16″ E). The fecal samples were frozen at −20 °C and transported back to the laboratory in Qufu within 24 h.

### 2.2. Bacterial Isolation

After the fecal samples arrived at the laboratory, the strains were immediately isolated using traditional microbial isolation and purification methods. The fecal samples were dissolved in sterile 0.85% saline and mixed well. The solution was then subjected to gradient dilution. Subsequently, 100 μL of the diluted homogenate was spread on LB agar plates and cultured overnight at 35 °C. Colonies with different morphological characteristics were selected and inoculated onto fresh LB agar plates. The isolated strains were purified by streaking at least three times on fresh LB agar plates, and finally, a single colony of strain BpQf-1 was isolated. The purified strains were preserved in 30% (*v*/*v*) glycerol solution at −80 °C for later use.

### 2.3. Morphological Observation of Pathogenic Bacteria

The preserved strains were cultured at 35 °C with a shaking speed of 150 rpm for 12 h, followed by three successive activation passages. The morphological characteristics of the pathogenic bacteria (colony morphology and pigmentation) were visually inspected on LB agar plates. The cell morphology was characterized using transmission electron microscopy (TEM) and negative staining (TEM). To prepare samples for TEM, the strains were cultured in LB liquid medium at 35 °C for 12 h. The bacterial suspension was then centrifuged at 6000 rpm for 5 min at 4 °C to obtain the precipitate. The precipitate was fixed in 2.5% glutaraldehyde for 2 h and washed three times with 0.1 M PBS, with a 10 min interval between each wash. The samples were then fixed in 1% osmium tetroxide at 40 °C for 4 h and washed three times with 0.1 M PBS, with a 10 min interval between each wash. The samples were dehydrated with ethanol solutions of 30%, 50%, 70%, and 80% for 10 min each, followed by two 10 min dehydrations with 100% ethanol. Finally, the samples were embedded in Epon812 epoxy resin and incubated at 37 °C, 45 °C, and 65 °C for 2 h each. Ultrathin sections were prepared using an Ultracut E machine and subsequently stained with lead nitrate and uranyl acetate for 15 min. The sections were observed under a JEM 1200EX transmission electron microscope (JEOL, Akishima, Tokyo, Japan).

To prepare negatively stained samples, the precipitate was prepared following the method used for TEM sample preparation. The precipitate was soaked in 3.0% glutaraldehyde for 10 min. A small drop of the suspension was placed on a carbon-coated copper grid for 3 min, then dried at room temperature for 15 min. The samples were examined by TEM using a JEM (JEOL, Akishima, Tokyo, Japan) instrument.

The absorbance of the bacterial suspension at 600 nm (OD_600_) was measured every 2 h, and colony counting was simultaneously performed using the dilution plating method. A standard growth curve was plotted based on these measurements, following the method reported by Tsutsuki H et al. [19]. A linear regression model was applied in Origin 2021 to generate the standard growth curve and calculate the corresponding R^2^ value. To ensure data transparency and reproducibility, both the growth curve and the standard curve were established using three biological replicates and three technical replicates.

### 2.4. Characterization of the Pathogenic Bacterial Strain Under the Biolog Gen III Microtest System

The physiological and biochemical characteristics of the strains were determined using the Biolog Gen III Micro Plate System. The strains were cultured in LB liquid medium at 35 °C for 12 h. A single colony was picked using a sterile disposable inoculation cotton swab and inoculated into Suspension B. The turbidity meter was used to adjust the target cell concentration to 90–98% T. The bacterial suspension was added to all wells of the microplate at a volume of 100 µL per well. The microplate was covered and incubated at 33 °C for 24 h. Data were automatically collected and read at 600 nm using the Biolog identification system (Biolog Inc., Hayward, CA, USA) [20].

### 2.5. Amplification and Sequencing of the 16S rRNA Gene and Construction of a Phylogenetic Tree

The 16S rRNA gene of strain BpQf-1 was amplified by PCR using the universal primers 27F and 1492R. The PCR products were examined by electrophoresis on a 2% agarose gel. Purification of the amplified products was carried out using the AxyPrep DNA Gel Extraction Kit (Axygen Biosciences, Union City, CA, USA), according to the manufacturer’s instructions. Bidirectional Sanger sequencing was performed on the purified 16S rRNA gene fragments. The similarity of strain BpQf-1 to its closely related strains was calculated according to the EzBioCloud server [21], and a phylogenetic tree was constructed based on the 16S rRNA of the related strains provided in the database. The phylogenetic tree was constructed using MEGA version 12 based on the neighbour-joining (NJ) method [22], and the Jukes-Cantor model [23] was selected. The robustness of each topology was checked by 1000 bootstrap replications.

### 2.6. Genome Sequencing and Analysis of Average Nucleotide Identity (ANI)

The EZ-10 Spin Column Bacterial Genomic DNA Isolation Kit (B610423-0050, Sangon Biotech, Shanghai, China) was used to extract genomic DNA from strain BpQf-1. Sequencing libraries were generated using the TruSeq DNA Sample Preparation Kit (Illumina, San Diego, CA, USA) and the Template Prep Kit (Pacific Biosciences, Menlo Park, CA, USA). The genome sequencing was then performed by Personal Biotechnology Company (Shanghai, China) by using the Pacbio Sequel II and the Illumina Novaseq platform. Data assembly was proceeding after adapter contamination removal and data filtering by using AdapterRemoval [24] and SOAPec [25]. The filtered reads were assembled by SPAdes [26] and A5-miseq [27] to construct scaffolds and contigs. Flye (v2.4, Cambridge, MA, USA) [28] and Unicycler (v0.4.7, Melbourne, VIC, Australia) [29] were used to assemble the data obtained by Nanopore platform sequencing. Subsequently, all assembled results were integrated to generate a complete sequence. Finally, the genome sequence was acquired after the rectification by using pilon (v1.22, Cambridge, MA, USA) [30].

To further understand the resolution of BpQf-1 with other closely related species strains in terms of evolutionary relationships, we conducted Average Nucleotide Identity (ANI) analysis. In prokaryotic taxonomy, the primary use of ANI is for species demarcation, with a critical value of approximately 95–96% typically being employed [31].

Subsequently, functional annotation of the gene functions of proteins based on their coding genes was performed by the Cluster of Orthologous Groups of proteins (COG) [32], Gene Ontology (GO) [33], and the Kyoto Encyclopedia of Genes and Genomes (KEGG) [34]. Prediction of virulence factors and resistance genes through the Virulence Factor Database (VFDB) [35] and the Comprehensive Antibiotic Resistance Database (CARD) [36].

### 2.7. Comparative Genomic Analysis

The complete genomes of two other strains of *B. paranthracis* and strains of *Bacillus paramycoides* and *Bacillus cereus* were selected from the National Center for Biotechnology Information (NCBI) GenBank database for comparative genomic analysis. The analysis included gene family, collinearity, and core/pan-genome analysis. Gene families were constructed using genes from closely related species and all genes from the target strains. After aligning the protein sequences with BLAST (v2.17.0), redundant sequences were removed using Solar (v2.0.4, Shenzhen, China). The aligned results were then subjected to gene family clustering using the Hcluster (v0.5.0, Shenzhen, China), which is based on the TreeFam clustering method. The clustered gene families were aligned using the Muscle (v3.8.1551, San Diego, CA, USA) [37,38]. The genome sequences of the target strains were aligned with the reference strains using the MUMmer (v4.0.0, Baltimore, MD, USA) [39] to construct a two-dimensional collinearity map. For core and pan-genome analysis, the CD-HIT (v4.8.1, Rockville, MD, USA) was used to cluster the protein gene sets of all strains to be analyzed. The final clustered gene set was considered as the pan-genome set, and sequences present in all samples were extracted as the core-genome set.

### 2.8. Mouse Lethality Assay

The experiment used SPF-grade Kunming Mice. These mice were 3 weeks old, with 9 males and 9 females, totaling 18 mice. They were purchased from Shandong Pengyue Laboratory Animal Technology Co., Ltd (Zhangqiu, Jinan, Shandong, China). The KM mice were divided into four experimental groups and two control groups, with three mice in each group. Each mouse in the experimental group was intraperitoneally injected with 0.1 mL of bacterial suspension, while each mouse in the control group was injected with an equal volume of sterile LB medium. The preserved strains were cultured at 35 °C for 12 h and activated for three consecutive generations. The bacterial suspension concentration was adjusted to 1 × 10^8^ CFU/mL, which was used as the challenge dose. The clinical changes of the mice were continuously observed within 12 h after the challenge, and finally, the pathological dissection results of the experimental and control groups were compared. Their lung and small intestine tissues were collected for hematoxylin and eosin (HE) staining, which was carried out by Wuhan Servicebio Co., Ltd (Wuhan, Hubei, China).

## 3. Results

### 3.1. Morphological and Staining Characteristics Observation and Growth Properties Study of Strain BpQf-1

**Figure 1:**
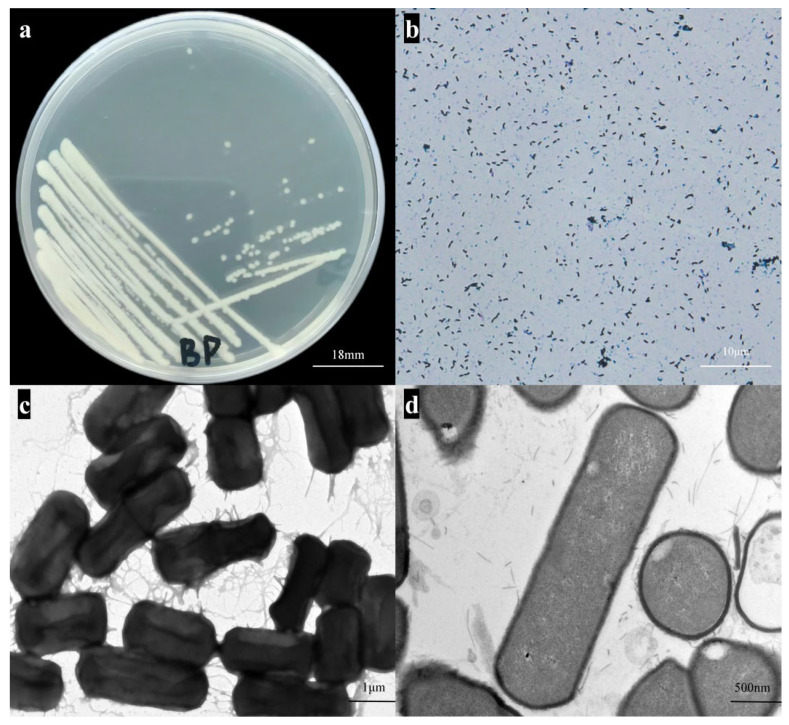
Strain Qf-1 isolated from mink feces. (**a**) Morphology of strain BpQf-1 on LB agar plates (bar = 18 mm). (**b**) Results of Gram staining for strain BpQf-1 (bar = 10 µm). (**c**) Morphology of strain BpQf-1 observed by negative staining SEM (bar = 1 µm). (**d**) Morphology of strain BpQf-1 observed by ultrathin section TEM (bar = 500 nm).

**Figure 2:**
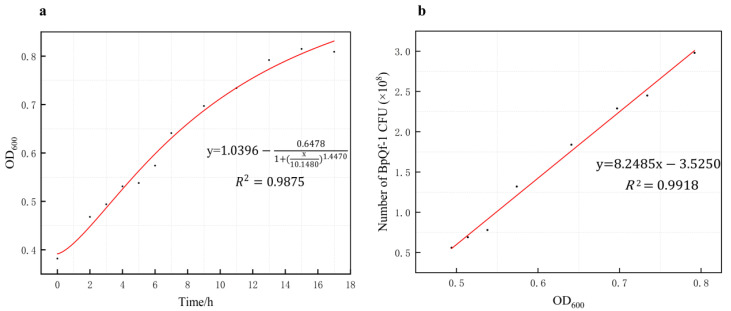
Growth curve and the concentration standard curve of strain BpQf-1. (**a**) The growth curve of strain BpQf-1. (**b**) The concentration standard curve of strain BpQf-1.

The colony morphology of strain BpQf-1 on LB agar appears as milky-white, circular dots with a moist surface and neat edges, forming nearly round single colonies (Figure 1a). Gram staining reveals that the strain is stained blue-purple (Figure 1b), indicating that BpQf-1 is a Gram-negative bacillus. Transmission electron microscopy (TEM) shows that BpQf-1 is rod-shaped, with a width of 1.0–1.5 μm and a length of 2.0–3.0 μm (Figure 1c, d). The growth curve of BpQf-1 indicates that the strain is in the logarithmic growth phase from 2 to 14 h and enters the stationary phase after 14 h (Figure 2a). By counting colonies at different growth times, a linear equation representing the relationship between OD_600_ and bacterial colony-forming units (CFU) was obtained (Figure 2b).

### 3.2. Biochemical Characterization of BpQf-1 Using Biolog Gen III Microtest System

In total, 94 different biochemical tests were performed on strain BpQf-1 (Table 1). It was found that the strain showed positive reactions to 43 (45.75%) of the tests and weakly positive reactions to nine (9.57%). The strain was able to utilize nine monosaccharides (Dextrin, d-Maltose, d-Trehalose, β-Methyl-d-Glucoside, n-Acetyl-d-Glucosamine, N-Acetyl-β-d-Mannosamine, d-Fructose, d-Fucose, Inosine), two hexose phosphates (d-Glucose-6-PO4, d-Fructose-6-PO4), seven hexosamines (Gelatin, l-Alanine, l-Arginine, l-Aspartic Acid, l-Glutamic Acid, l-Histidine, l-Serine), and 21 carboxylic acids, esters, and fatty acids (d-Galacturonic Acid, d-Gluconic Acid, Methyl Pyruvate, d-Lactic Acid Methyl Ester, d-Malic Acid, Propionic Acid, etc.) as carbon sources. Negative reactions were observed for 42 (44.68%) of the tests.

### 3.3. Molecular Identification and Phylogenetic Analysis

**Figure 3:**
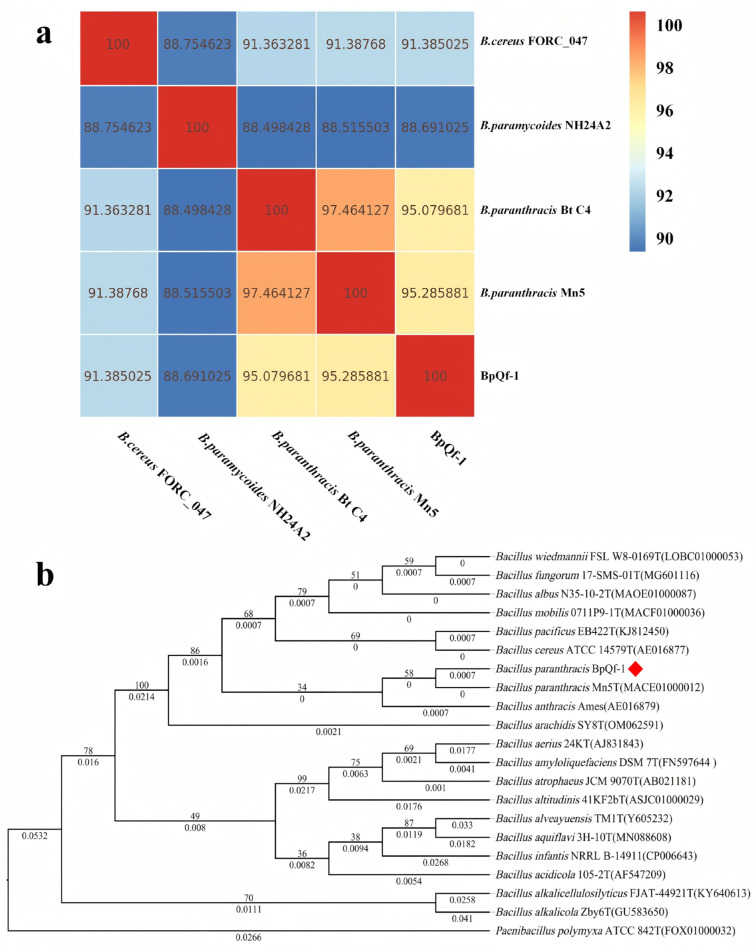
Molecular identification and phylogenetic analysis of the strain BpQf-1. (**a**) Heat map of ANI. (**b**) The phylogenetic tree constructed based on 16S rRNA, the strain investigated in this study is indicated by a red diamond.

A phylogenetic tree was constructed based on the 16S rRNA of BpQf-1 for evolutionary analysis (Figure 3b). The results showed that BpQf-1 is most closely related to *B. paranthracis*. Meanwhile, the ANI heatmap analysis (Figure 3a) indicated that the ANI values between BpQf-1 and the two strains of *B. paranthracis* were both greater than 95%, suggesting a close evolutionary relationship among them.

### 3.4. Genomic Features of Strain BpQf-1

As shown in Table 2, the clean reads of *B. paranthracis* Qf-1 were 1261 bp. The genome size of *B. paranthracis* Qf-1 was 5,276,703 bp with a GC content of 36.13%. A total of 184 non-coding RNAs were identified by sequencing, including 105 tRNAs, 14 each of 5s rRNA, 16s rRNA, and 23s rRNA, and 37 sRNAs. There were approximately 5334 protein-coding genes, and 3822, 2971, 2923, and 2592 genes were annotated against the Cluster of Orthologous Groups of proteins (COG), Kyoto Encyclopedia of Genes and Genomes (KEGG), Gene Ontology (GO), and Swiss-Pro databases, respectively. In addition, their 7, 286, 4, and 593 genes were annotated against the Clustered Regularly Interspaced Short Palindromic Repeats (CRISPRs) (Table S1), Virulence Factor Database (VFDB) (Table S2), Comprehensive Antibiotic Resistance Database (CARD) (Table S3), and type III secretion system (T3SS) (Table S4), respectively.

**Figure 4:**
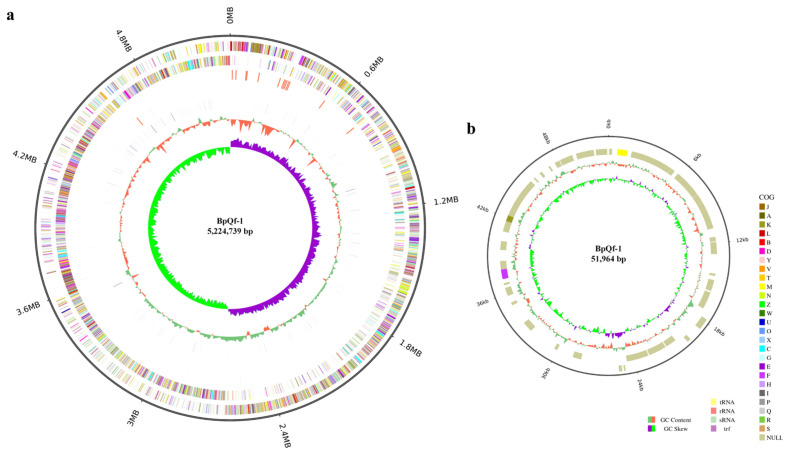
Genome map of the BpQf-1 chromosome. (**a**) Chromosome genome map, the circles represent (from outer to inner) genome size, positive strand gene COG annotation, reverse strand gene COG annotation, positive strand ncRNA distribution, reverse strand ncRNA distribution, repeat, GC%, GC-SKEW. (**b**) Plasmid genome map, the circles represent (from outer to inner) genome size, positive strand gene COG annotation, reverse strand gene COG annotation, GC%, GC-SKEW.

Based on the whole-genome sequencing results, genome completion maps were separately drawn for the chromosome sequence and the plasmid sequence. The genome size of strain BpQf-1 is 5.27 Mb, comprising one chromosome (5,224,739 bp) (Figure 4a) and one plasmid (51,964 bp) (Figure 4b). In the chromosome map, the outermost circle represents the size of the genome, followed from outside to inside by the COG functional annotation results, and then the distribution of ncRNAs. In the plasmid plot, the genome size, COG functional annotation, and GC distribution are shown from outside to inside.

### 3.5. Functional Annotation of Strain BpQf-1

**Figure 5:**
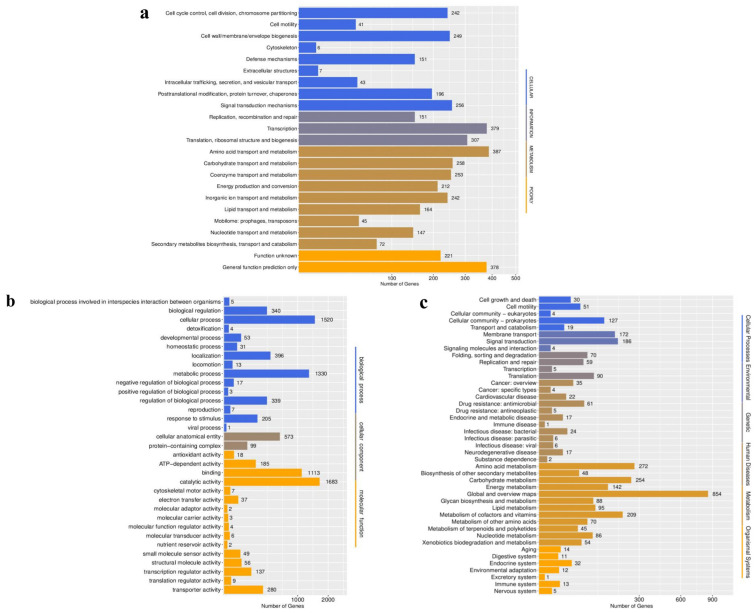
Statistical legend of gene annotation classification of BpQf-1. (**a**) The COG annotation of the strain BpQf-1. (**b**) The GO annotation of the strain BpQf-1. (**c**) The KEGG annotation of the strain BpQf-1.

A total of 4407 BpQf-1 coding genes were annotated by COG functional categories (Figure 5a). Among them, 242 genes are responsible for cell cycle control, cell division, and chromosome partitioning (5.49%). A total of 41 genes are involved in cell motility (0.95%), 249 genes participate in the formation of cell wall/membrane/envelope biogenesis (5.65%), six genes are involved in cytoskeleton generation (0.14%), 151 genes are involved in defense mechanisms (3.43%), seven genes are involved in the generation of extracellular structures (0.16%), 43 genes are involved in intracellular trafficking, secretion, and vesicular transport (0.98%), 196 genes are involved in posttranslational modification, protein turnover, chaperones (4.45%), 256 genes are involved in signal transduction mechanisms (5.81%), 151 genes are involved in replication, recombination and repair (3.43%), 379 genes are involved in transcription (8.60%), 307 genes are involved in translation and ribosomal structure and biogenesis (6.97%), 387 genes are responsible for amino acid transport and metabolism (8.78%), 258 genes are responsible for carbohydrate transport and metabolism (5.85%), 253 genes are responsible for coenzyme transport and metabolism (5.74%), 212 genes are involved in energy production and conversion (4.81%), 242 genes are responsible for inorganic ion transport and metabolism (5.49%), 164 genes are responsible for lipid transport and metabolism (3.72%), 45 genes are involved in mobilome: prophages, transposons (1.02%), 147 genes are responsible for nucleotide transport and metabolism (3.34%), 72 genes are involved in secondary metabolites biosynthesis, transport and catabolism (1.63%), 378 genes are involved in general functions (8.58%), and 221 genes encode proteins with unknown functions (5.01%).

The BpQf-1 genes, with a total of 8527 annotated by the Gene Ontology (GO) system, describe 4264 biological processes, 672 cellular components, and 3591 molecular functions (Figure 5b). Among the genes involved in biological processes, 340 genes are involved in biological regulation (3.99%), 1520 genes are involved in cellular processes (17.83%), 396 genes are involved in localization (4.64%), 1330 genes are involved in metabolic processes (15.60%), 339 genes are involved in regulation of biological processes (3.98%), and 205 genes are involved in response to stimuli (2.40%). Among the genes involved in cellular components, 573 genes are involved in cellular anatomical entity (6.72%), and 99 genes are involved in protein-containing complex (1.16%). Among the genes involved in molecular functions, 185 genes are involved in ATP-dependent activity (2.17%), 1113 genes are involved in binding (13.05%), 1683 genes are involved in catalytic activity (19.74%), and 280 genes are involved in transporter activity (3.28%).

The KEGG enrichment analysis annotated a total of 3322 genes of BpQf-1, among which 231 genes are involved in cellular processes (6.95%), 362 genes are involved in environmental information processing (10.90%), 224 genes are involved in genetic processes (6.74%), 200 genes are involved in human diseases (6.02%), 2217 genes are involved in metabolism (66.74%), and 88 genes are involved in biosynthetic systems (2.65%) (Figure 5c).

### 3.6. Analysis of Virulence Factors and Antibiotic Resistance Genes of BpQf-1

In the genome of BpQf-1, a total of 286 potential virulence factor genes were identified, including 238 predicted virulence factors (83%) and 48 verified virulence factors (17%). Among the verified virulence factors, nine virulence factor genes had a similarity of over 70% (Table 3). According to the database comparison results, there were two Nutritional/Metabolic factors: *AsbD* and *AsbF*; four Effector delivery system genes: *EsxB*, *EsxL*, *EssC*, and *BAS_RS10600*, which are associated with type VII secretion system proteins; two Stress survival-related genes: *ClpP* and *ClpC*; and one Exoenzyme-related gene: *InhA*.

Comparison with the CARD database revealed four antibiotic resistance genes (Table 4). The gene *Bla2* is associated with resistance to carbapenems, cephalosporins, and penams; *FosB* confers resistance to fosfomycin; and *MphL* is related to resistance to macrolide antibiotics. These genes function by inactivating the corresponding antibiotics. Additionally, the gene *MCR-4.1*, which is associated with resistance to peptide antibiotics, functions by altering the antibiotic target.

### 3.7. Collinearity Analysis

**Figure 6:**
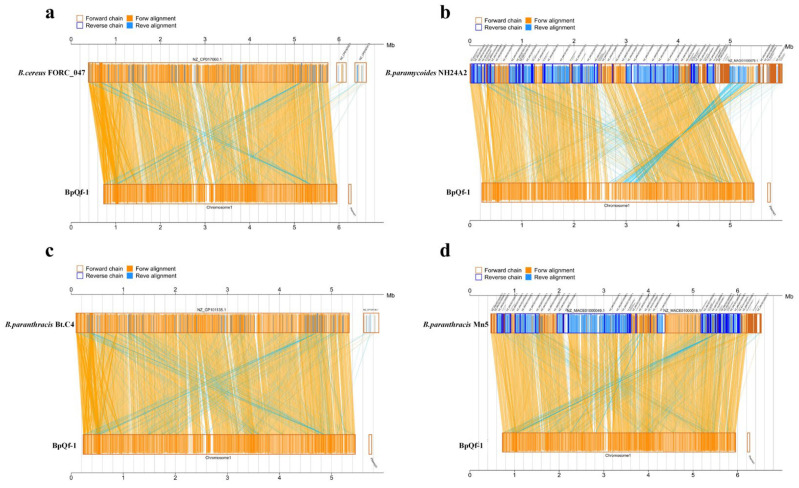
Collinearity analysis. (**a**) Collinearity plot of BpQf-1 and *B. cereus* FORC_047. (**b**) Collinearity plot of BpQf-1 and *B. paranycoides* NH24A2. (**c**) Collinearity plot of BpQf-1 and *B. paranthracis* Bt.C4. (**d**) Collinearity plot of BpQf-1 and *B. paranthracis* Mn5.

We performed collinearity analysis between the target genome and four reference genomes. The results showed that the genome of BpQf-1 has high homology with *B. paranthracis* (Figure 6c,d). In the collinearity map with *Bacillus paramycoides* (Figure 6b), there were larger insertions and lower homology, which is consistent with the ANI analysis results. Compared with BpQf-1, *B. paranthracis* Mn5 has partial translocations and inversions, but *B. paranthracis* Bt C4 has almost no chromosomal variations and shows overall conservation.

### 3.8. Core Genome and Pan Genome Analysis

**Figure 7:**
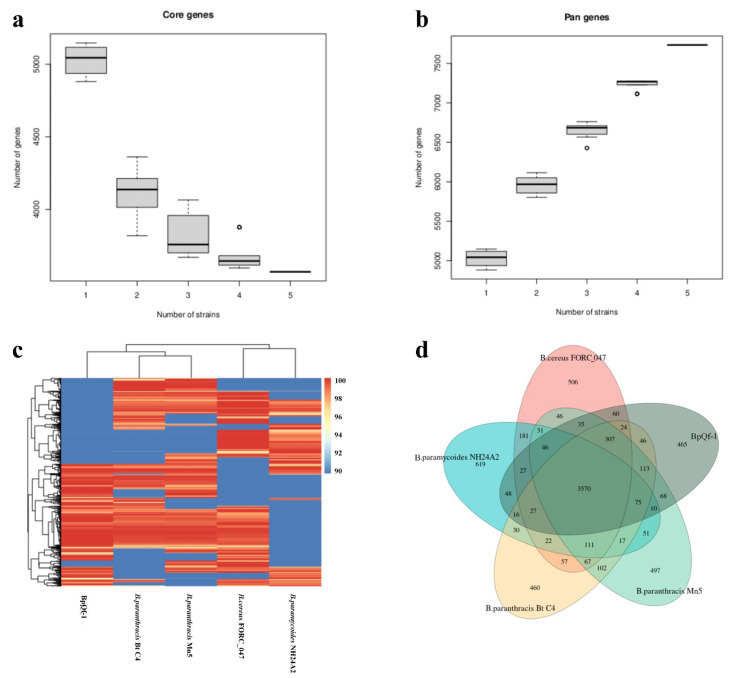
Compare the genomic differences between BpQf-1 and four other strains through core pan-genome analysis. (**a**) Core gene rarefaction curve, the circle represents outliers data. (**b**) Pan gene rarefaction curve, the circle represents outliers data. (**c**) Dispensable gene heat map, the left side shows the clustering tree of dispensable genes, the top shows the clustering tree of samples, the middle shows the gene similarity heat map, with different coverage levels represented by different colours, and the legend in the upper right corner shows the correspondence between colours and coverage levels. (**d**) Pan genome Venn diagram, the data for each region represents the number of clusters that appear in the samples in that region only. A cluster represents a group of genes with greater than 50% similarity and a sequence length difference of less than 0.3.

Core and pan genome analysis was conducted on five bacterial strains. The core genome comprises 3330 genes, which were subjected to COG enrichment analysis (Figure S1). The main categories include: 199 genes involved in cell cycle regulation, cell division, and chromosome segregation (5.98%), 264 genes involved in translation, ribosomal structure, and biosynthesis (7.93%), 312 genes involved in amino acid transport and metabolism (9.37%), and 296 genes involved in general functions (8.89%). Additionally, 111 genes were unique to BpQf-1 (Table S5). As the number of bacterial strains increased, the number of core genes decreased, while the pan-genome exhibited the opposite trend (Figure 7a,b). The increase in the number of pan-genes with the addition of more strains indicates that the genome of BpQf-1 is more diverse and contains a greater number of unique genes.

### 3.9. Pathological Analysis to Explore Strain Pathogenicity

**Figure 8:**
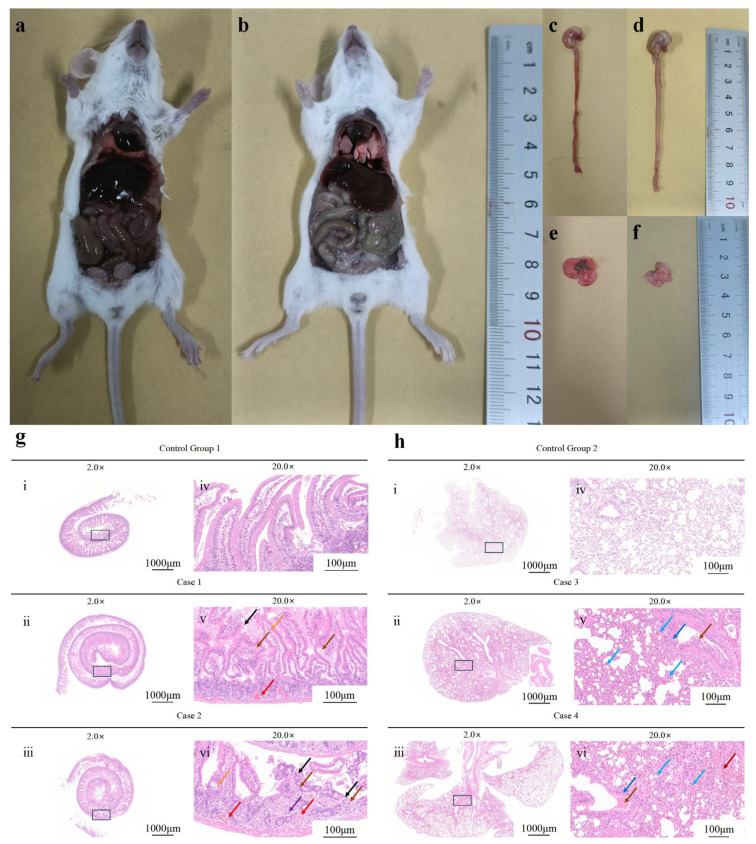
Pathological observations of pathogenicity experiments in mice. (**a**) Anatomy of organs in mice injected intraperitoneally with Qf-1. (**b**) Organ anatomy of mice injected intraperitoneally with LB medium. (**c**) Small intestine injected intraperitoneally with Qf-1. (**d**) Small intestine injected intraperitoneally with LB medium. (**e**) Lungs with intraperitoneal injection of Qf-1. (**f**) Lungs injected intraperitoneally with LB medium. (**g**) Small intestine HE staining results. (**h**) Lungs HE staining results. The control group 1, 2 was administered LB liquid medium via intraperitoneal injection, while case 1 to case 4 were the experimental groups that received intraperitoneal injection of strain Qf-1. Orange arrow: Separation of the intestinal villus epithelium from the lamina propria. Black arrow: Numerous mucosal epithelial cells have sloughed off. Brown arrow: Presence of a small amount of eosinophilic material. Red arrow: Mild interstitial vascular congestion in the lamina propria. Purple arrow: Infiltration of a small number of inflammatory cells, mainly lymphocytes. Blue arrow: Numerous granulocytes infiltrating the alveolar walls. Dark blue arrow: Edema of a small number of bronchial mucosal epithelial cells. Dark red arrow: Focal hemorrhage. Bar: (**g**) (**i**–**iii**) and (**h**) (**i**–**iii**) = 1000 µm; Bar: (**g**) (**iv**–**vi**) and (**h**) (**iv**–**vi**) = 100 µm.

Mice in the experimental group exhibited signs of listlessness and reduced appetite within 2 h after intraperitoneal inoculation with the BpQf-1 strain. They huddled in the corners and experienced shivering. All mice in the experimental group died within 12 h, while no abnormalities were observed in the control group. On necropsy of the deceased mice from the experimental group, significant hemorrhage and congestion were observed in the lungs and intestines, along with enlarged spleen and liver (Figure 8a). In contrast, no significant pathological changes were found in the control group mice.

Histopathological sections of the intestines and lungs of the deceased experimental group mice revealed small-scale erosion of the intestinal tissue, a reduced number of intestinal glands, and a large number of shed mucosal epithelial cells. The lamina propria showed interstitial vascular congestion. In the lung tissue, the alveolar septa were widened, and the alveolar walls were moderately thickened. A few alveoli showed compensatory expansion. Additionally, there was edema of a few bronchial mucosal epithelial cells, interstitial vascular congestion, and alveolar hemorrhage (Figure 8b). These findings indicate the strong pathogenicity of BpQf-1.

## 4. Discussion

The *Bacillus cereus* group, which includes *B. paranthracis*, comprises spore-forming, Gram-positive bacilli commonly associated with diarrheal or emetic food poisoning [40]. *Bacillus cereus*, the type species of the group, is a conditional human pathogen capable of causing bacteremia [41], respiratory, urinary tract infections, gastrointestinal diseases, and meningitis [42,43]. While most *Bacillus* spp. are non-pathogenic in healthy individuals, they can become opportunistic pathogens in immunocompromised hosts [44].

The *B. paranthracis* strain was first isolated and identified from sediments and seawater collected from marine environments by Liu et al. in 2017 [11]. Through experiments using Luria-Bertani (LB) medium, the morphology, physiology, and chemotaxonomy of the strain were characterized, and its 16S rRNA gene sequence was obtained. It was determined that the strain belongs to the *Bacillus cereus* group. Subsequently, through genome sequencing and calculation of average nucleotide identity (ANI) values, Liu et al. confirmed that the nine strains they discovered, including *B. paranthracis*, were new species. This work not only enriched the composition of the *Bacillus cereus* group but also laid the foundation for the study of *B. paranthracis*, making a significant contribution to the field. In this study, strain Qf-1 was isolated from the feces of minks. Through morphological observation, physiological and biochemical identification, 16S rRNA gene sequence analysis, and ANI analysis, Qf-1 was named BpQf-1 (*Bacillus paranthracis* Qf-1). *B. paranthracis* has been identified as a conditional pathogen that can cause foodborne diarrhea and emesis in humans [17]. In 2024, a foodborne outbreak caused by *B. paranthracis* was reported in Huzhou, Zhejiang, China [18]. In 2020, a waterborne outbreak of *B. paranthracis* was documented in a suburban school in China, highlighting its pathogenic potential and complexity [14]. To date, there have been few reports of diseases caused by *B. paranthracis*, especially in minks. This study provides the first in-depth analysis of a *B. paranthracis* strain, designated as Qf-1, isolated from minks.

Subsequently, Matson et al. isolated *B. paranthracis* from the blood of a fatal Ebola virus disease (EVD) case [12]. The isolate was identified by whole-genome sequencing and may have contributed to the patient’s bacteremia, complicating the disease. *B. paranthracis* was also isolated from pet reptiles in Poland [13]. Analysis of average nucleotide identity (ANI) values confirmed its identification as *B. paranthracis*. Subsequently, *B. paranthracis* was isolated for the first time from the surface of book pages [15]. This finding demonstrated the wide distribution of the strain in various environments. Studies on its motility, biofilm-forming ability, cytotoxicity, and enterotoxicity suggested the potential pathogenicity of *B. paranthracis* [15].

In this study, by comparing the pathogenicity factor database, we identified the presence of the *EsxB* and *EsxL* genes. These genes encode WXG100 proteins, which are typical substrates of the bacterial Type VII secretion system [45]. These effector proteins can disrupt the integrity of host cells, modulate inflammatory responses, and facilitate immune evasion by bacteria, thereby exacerbating disease progression. The regulation of proteins encoded by *AsbF* and *AsbD* is similar to the mechanism of siderophore biosynthesis in other bacteria. They chelate trivalent iron through the 3,4-isomer of 2,3-dihydroxybenzoic acid (3,4-DHBA). Siderophores are essential tools for bacteria to acquire iron, which is crucial for their full activity in the environment and for the expression of virulence in the host [46,47]. These mechanisms can lead to exacerbated pulmonary inflammation and disease progression, and may also intensify enteritis by disrupting the integrity of the intestinal barrier and activating the intestinal mucosal immune system. Research into these mechanisms will aid in the development of new therapeutic strategies against bacterial infections [48,49]. The virulence genes *ClpP* and *ClpC* are associated with the ability of bacteria to form biofilms [50]. Biofilm formation enhances the survival capacity of bacteria, allowing them to persist in various environments [51]. The virulence gene *InhA* enables bacteria to secrete a zinc metalloprotease that can hydrolyze antimicrobial proteins produced by insect hosts. This suggests that it may contribute to the overall virulence of bacterial strains carrying this virulence factor [52,53].

Currently, in our country, the traditional bacterial culture identification method has been combined with the 16S rRNA gene sequence molecular identification method to identify different pathogens. For the *Bacillus cereus* group, whole-genome sequencing and average nucleotide identity (ANI) are used to differentiate the genomes of species. Generally, if the ANI value between two genomes is ≥95%, they are considered to be the same species. In this study, the pathogen *Bacillus paranthracis* Qf-1 was isolated and identified, and its genome structure and function were analyzed using multi-omics technologies, enriching the information on conditional pathogens of the economically important farmed animal, the mink.

## 5. Conclusions

Mink is an important economic animal for breeding. Over time, mink farming has become increasingly susceptible to various bacterial infections, which pose a threat to the development of the mink breeding industry as well as to the health of humans and other animals. Currently, research on bacterial diseases in minks is still incomplete. Therefore, the isolation and identification of mink-derived pathogens are of great significance for the diagnosis and prevention of bacterial diseases in minks. In this study, the strain *Bacillus paranthracis* Qf-1 was isolated, purified, and identified from an individual mink. Through culturomics, 16S rRNA gene sequencing, whole-genome sequencing, and comparative genomic analysis, we investigated the genetic characteristics and functions to explore its potential pathogenicity. These research findings contribute to the establishment of a stable experimental model between pathogens and mink hosts, laying the foundation for further elucidating their pathogenicity and pathogenic mechanisms. This is of great significance for the diagnosis and prevention of bacterial diseases in mink in the future.

## Figures and Tables

**Table 1:** Characterization of strain BpQf-1 based on the Biolog Gen III microplate.

Positive Reaction with the Following Substrate/Test		
Dextrin	d-Galacturonic Acid	Acetic Acid
d-Maltose	l-Galactonic Acid Lactone	Formic Acid
d-Trehalose	d-Gluconic Acid	pH 6
β-Methyl-d-Glucoside	d-Glucuronic Acid	1% NaCl
N-Acetyl-d-Glucosamine	Glucuronamide	4% NaCl
d-Fructose ^1^	d-Saccharic Acid	8% NaCl
Glycerol	Methyl Pyruvate	1% Sodium Lactate
d-Glucose-6-PO4	d-Lactic Acid Methyl Ester	d-Serine
d-Fructose-6-PO4	l-Lactic Acid	Guanidine HCl
d-Serine	l-Malic Acid	Lithium Chloride
Gelatin	Bromo-Succinic Acid	Potassium Tellurite
l-Arginine	Tween 40	Aztreonam
l-Aspartic Acid	β-Hydroxy-d, l-Butyric Acid	Sodium Butyrate
l-Glutamic Acid	Acetoacetic Acid	
l-Serine	Propionic Acid	
**Weak Positive Reaction with the Following Substrate/Test**		
N-Acetyl-β-D Mannosamine	L-Alanine	Citric Acid
D-Fucose	L-Histidine	α-Keto-Glutaric Acid
Inosine	Mucic Acid	D-Malic Acid
**Negative Reaction with the Following Substrate/Test**		
D-Cellobiose ^1^	L-Fucose	pH 5
Gentiobiose	L-Rhamnose	Fusidic Acid
Sucrose	D-Sorbitol	Troleandomycin
D-Turanose ^1^	D-Mannitol	Rifamycin SV
Stachyose	D-Arabitol	Minocycline
D-Raffinose	myo-Inositol	Lincomycin
α-D-Lactose	D-Aspartic Acid	Niaproof 4
D-Melibiose	Glycyl-L-Proline	Vancomycin
D-Salicin	L-Pyroglutamic Acid	Tetrazolium Violet
N-Acetyl-D-Galactosamine	Pectin	Tetrazolium Blue
D-Cellobiose	L-Fucose	pH 5
N-Acetyl-Neuraminic Acid	Quinic Acid	Nalidixic Acid
α-D-Glucose	p-Hydroxy-Phenylacetic Acid	Sodium Bromate
D-Mannose ^1^	γ-Amino-Butryric Acid	
D-Galactosea ^1^	α-Hydroxy Butyric Acid	
3-Methyl Glucose	α-Keto-Butyric Acid	

^1^ Adapted from Liu [11].

**Table 2:** *B. paranthracis* whole-genome sequencing results statistics.

Characteristic	Genome	Characteristic	Gnome
Raw data (Mb)	1310	CRISPRs	7
Total reads	8,738,130	VFDB	286
Clean data (Mb)	1261	CARD	4
Genome size (bp)	5,276,703	T3SS	593
GC content (%)	36.13	Coding gene annotated	5334
Gene total size (bp)	4,409,133	Coding gene assigned to COG	3822
rRNA	42	Coding gene assigned to KEGG	2971
tRNA	105	Coding gene assigned to GO	2923
ncRNA	184	Coding gene assigned to Swiss Prot	2592
sRNA	37		

**Table 3:** Virulence factors in BpQf-1 genome.

Gene Name	Category	Description	Identify (%)
*AsbD*	Nutritional/Metabolic factor	petrobactin biosynthesis protein *AsbD*	100
*EsxB*	Effector delivery system	type VII secretion system protein *EsxB*	100
*EssC*	Effector delivery system	type VII secretion system protein *EssC*	99.7
*AsbF*	Nutritional/Metabolic factor	petrobactin biosynthesis 3-dehydroshikimate dehydratase *AsbF*	99.6
*BAS_RS10600*	Effector delivery system	type VII secretion system protein	99.3
*InhA*	Exoenzyme	immune inhibitor A metalloprotease	99.2
*EsxL*	Effector delivery system	type VII secretion system protein *EsxL*	97.2
*ClpP*	Stress survival	ATP-dependent *Clp* protease proteolytic subunit	78.2
*ClpC*	Stress survival	endopeptidase *Clp* ATP-binding chain C	77

**Table 4:** Antibiotic resistance genes in BpQf-1 genome.

Gene Name	Antibiotics	Resistance Mechanism	AMR Gene Family	Identify (%)
*MCR-4.1*	peptide antibiotic	antibiotic target alteration	MCR phosphoethanolamine transferase	100
*Bla2*	carbapenem; cephalosporin; penam	antibiotic inactivation	subclass B1 *Bacillus anthracis Bla* beta-lactamase	95.93
*FosB*	fosfomycin	antibiotic inactivation	fosfomycin thiol transferase	89.86
*MphL*	macrolide antibiotic	antibiotic inactivation	macrolide phosphotransferase (MPH)	88.08

## Data Availability

The original data presented in the study are openly available in a publicly accessible repository. The raw sequence data reported in this paper were deposited in the Genome Sequence Archive (Genomics, Proteomics and Bioinformatics 2021) in the National Genomics Data Center (Nucleic Acids Res 2022), China National Center for Bioinformation/Beijing Institute of Genomics, Chinese Academy of Sciences, which are publicly accessible at https://ngdc.cncb.ac.cn/gsa, access on 29 June 2025. CRA028417 (short-read) and CRA028862 (long-read) are the accession numbers of the genome data of *B.paranthracis* Qf-1; meanwhile, CRA028421 is the accession number of the 16S rRNA gene sequence.

## Supplementary Materials

The following supporting information can be downloaded at: https://www.mdpi.com/article/10.3390/microorganisms13092106/s1. Table S1: The analysis of CRISPR of BpQf-1. Table S2: The analysis of vfdb of BpQf-1. Table S3: The analysis of card of BpQf-1. Table S4: The analysis of T3SS of BpQf-1. Table S5: The analysis of unique gene of BpQf-1. Figure S1: COG enrichment analysis of core genes of BpQf-1.
