# Supplementary figures and images for: Whole-Genome Analysis of Bacillus paranthracis Qf-1 Isolated from Mink (Neogale vison)
Source: Microorganisms. 2025 Sep 9;13(9):2106. doi: 10.3390/microorganisms13092106 (PMC12472461; doi:10.3390/microorganisms13092106)

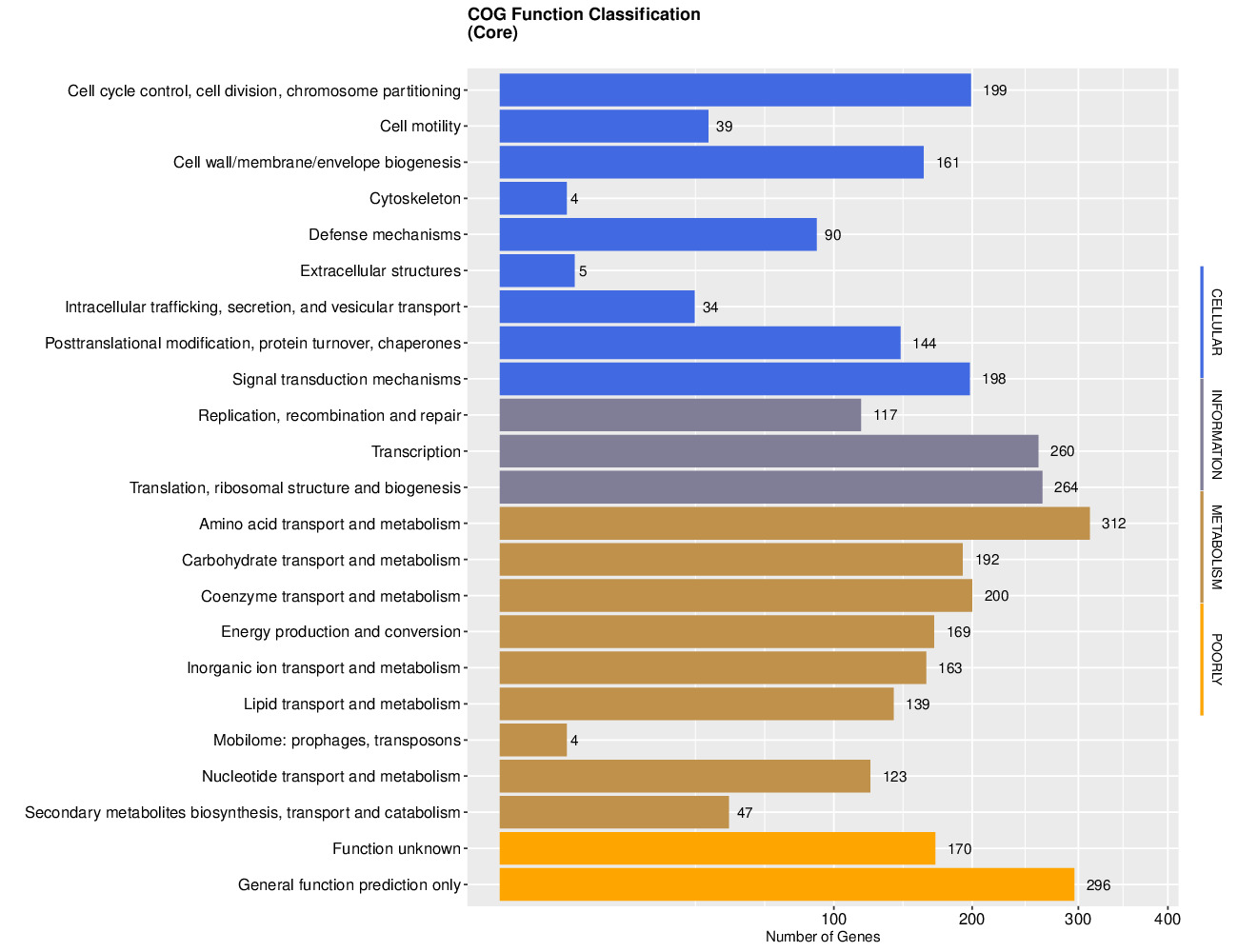

Supplement: Supplementary file 1 [file microorganisms-13-02106-s001.zip › Figure S1 COG enrichment analysis of core genes of BpQf-1.jpg]
